# Host Plant Specificity in Web-Building Spiders

**DOI:** 10.3390/insects14030229

**Published:** 2023-02-24

**Authors:** Thomas Hesselberg, Kieran M. Boyd, John D. Styrsky, Dumas Gálvez

**Affiliations:** 1 Department for Continuing Education, University of Oxford, Oxford OX1 2JA, UK; 2 Department of Biology, University of Oxford, Oxford OX1 3SZ, UK; 3 School of Biological Sciences, Queen’s University Belfast, Belfast BT7 1NN, UK; 4 Department of Biology, University of Lynchburg, Lynchburg, VA 24501, USA; 5 Coiba Scientific Station, Panama City 0843-01853, Panama; 6 Programa Centroamericano de Maestría en Entomología, Universidad de Panamá, Panama City 0824, Panama; 7 Smithsonian Tropical Research Institute, Panama City P.O. Box 0843-03092, Panama

**Keywords:** spider–plant interactions, swollen thorn acacias, carnivorous plants, orb-web spiders, host recognition, plant volatiles

## Abstract

**Simple Summary:**

Many invertebrates interact and are associated with plants in nature. However, despite their abundance and ecological importance, our knowledge of spiders and their associations with plants is limited. Here, we review what we currently know about spider–plant interactions and associations, with a focus on web-building spiders. This includes an overview of the most prominent interactions non-web-building and web-building spiders have with plants, followed by examples of the specific web-building spider–plant associations we know of, where especially the Acacia–*Eustala* association observed in Panama is interesting. We also review the plausible mechanisms for host plant location and finally present some ideas for future research.

**Abstract:**

Spiders are ubiquitous generalist predators playing an important role in regulating insect populations in many ecosystems. Traditionally they have not been thought to have strong influences on, or interactions with plants. However, this is slowly changing as several species of cursorial spiders have been reported engaging in either herbivory or inhabiting only one, or a handful of related plant species. In this review paper, we focus on web-building spiders on which very little information is available. We only find well-documented evidence from studies of host plant specificity in orb spiders in the genus *Eustala*, which are associated with specific species of swollen thorn acacias. We review what little is known of this group in the context of spider–plant interactions generally, and focus on how these interactions are established and maintained while providing suggestions on how spiders may locate and identify specific species of plants. Finally, we suggest ideas for future fruitful research aimed at understanding how web-building spiders find and utilise specific plant hosts.

## 1. Introduction

Plants are a vital resource for many animals that use them for food, shelter or protection. The best known plant–animal interactions involve insects and include negative interactions, such as herbivory, and positive interactions, such as pollination, and other mutualistic interactions. In many of these interactions, the insect shows specificity in that it only interacts with one, or a couple of plant species. These examples can be tightly co-evolved and include the food-for-protection mutualism between ants and swollen thorn acacias, where a specific species of ant is paired with a specific species of acacia [1], the extreme specificity of fig wasp pollinators to particular fig species hosts [2], and the specialisation of small groups of orchids to one species of bee pollinator, such as the South African guild of orchids (Coryciinae) exclusively relying on the oil-collecting bee (*Rediviva peringueyi*) for pollination [3].

Insects, as outlined above, and other arthropods, such as herbivorous and mutualistic mites [4], are well known for developing close associations with plants. Spiders, however, are usually thought of as generalist predators that only use vegetation indiscriminately for shelter or as a substrate for their webs. A study on a temperate grassland spider community, for example, showed that while some individual spider species showed a weak preference for a narrow range of host plants, the overwhelming preference was for tall and stable vegetation structures and not individual plant species [5]. Recently, the long-held notion that spiders have limited interactions with the vegetation in their surroundings have been challenged, especially by the surprising discovery that some species of spiders, and the first instars of web-building spiders in particular, rely on nectar, pollen and Beltian bodies as a significant component of their diets [6,7,8]. This prompted a review of spider–plant interactions in general, which revealed associations with plants across a much larger range of spider families than previously thought [9].

Very limited research is available on spiders that construct aerial webs, which predominantly consist of sheet-webs by members of the family Linyphiidae, tangle webs by members of the family Theriididae, and orb webs by members of the families Araneidae and Tetragnathidae. As the function of the webs to some degree depends on the substrate to which they are attached, it could be argued that they are more dependent on the correct choice of plant, and therefore, potentially should be more discerning than cursorial spiders. A relatively newly described species of linyphiid, *Laetesia raveni*, from Australia appears to exclusively build its webs on two thorny plant species, *Calamus muelleri* and *Solanum inaequilaterum* [10]. Similarly, one genus of araneid spiders, *Eustala*, seems a promising candidate for more in-depth research as several studies show close associations to individual species of acacias in the genus *Vachellia* [11,12]. These acacia species are in a mutualistic relationship with protective *Pseudomyrmex* ants, and the *Eustala* spiders probably associate closely with the acacias to exploit the ant–acacia mutualism for enemy-free space [13].

Another largely unresolved question is how spiders locate and identify their host plants. Insects generally locate their host plants using chemical cues from wind-dispersed plant volatiles [14,15]. In ant–plant associations, ants identify their mutualistic partner by chemical cues emitted from the plant [16]. However, the distance to which they rely on plant volatiles, or random searches for the location of host plants remains unclear. On the one hand, *Pheidole minutula* used plant volatiles to correctly locate their host plant *Maieta guianensis* during choice tests over distances of 15 cm in Y-maze experiments in the laboratory [17], while on the other hand, *Crematogaster* ants recognise their host *Macaranga* species only by direct contact with chemical compounds on the stem surface of saplings [18]. Spiders are also known to use chemical cues during mating behaviour [19], such as males using cues from silk to locate and evaluate females [20], and they use them to detect potential prey [21]. In addition, there are a few examples of spiders using chemical cues from plants, including two species of crab spiders in the genus *Thomisus* that were attracted to the clove oil flower fragrance [22] and the nectivorous spider *Hibana futilis*, which uses plant volatiles to recognise and potentially locate nectar sources [6].

The main aim of this review paper is to review the limited data we have on host plant specificity in web-building spiders and to contrast it with what is known from cursorial spiders. Secondarily, we review the limited literature on how web-building spiders identify and find web-building locations, including suitable plants, using the above-mentioned *Eustala* orb spiders as a model system. We hope to stimulate further research by identifying significant gaps and outlining promising experimental approaches to plug some of these gaps.

## 2. Spider–Plant Associations

In this section, we provide a brief overview of some of the best described examples of close spider–plant associations for both cursorial (i.e., non-web-building) and web-building spiders. This has been recently reviewed by Vasconcellos-Neto et al. [9], but here we update with newer references focussing predominantly on web-building spiders and link the topic to host plant locations in general and the *Eustala*–acacia–ant system in particular.

### 2.1. Cursorial Spiders

Bromeliads and other rosette-structured plants have a complex, three-dimensional architecture that presents a valuable microhabitat for a number of species [23], particularly members of Salticidae [24,25,26]. The best studied cursorial spider–plant association, and one of the few species-specific examples, is that of the bromeliad specialist *Psecas chapoda* and *Bromelia balansae*. Through a series of studies by Romero and Vasconcellos-Neto [27,28,29], *P. chapoda* was found exclusively on *B. balansae* across a large geographic range [26] (Table 1). Whilst *B. balansae* provides *P. chapoda* with a favourable microhabitat and microclimate, *P. chapoda* has been reported to contribute to the nutrition of *B. balansae* through the absorption of nitrogen from spider faeces deposited on the leaves of the bromeliad [30]. Romero et al. [31,32] evidenced that this interaction was indeed mutualistic as the leaves of *B*. *balansae* grew larger in the presence of *P. chapoda*. 

Some Thomisidae crab spiders, which have been documented as obligate *Nepenthes* pitcher-plant dwellers (Table 1), have likewise been reported to assist their host plant with nitrogen acquisition. The specialised leaves of pitcher-plants, which are used to attract, trap, and digest prey [33,34], also provide suitable microhabitats for the crab spiders *Misumenops nepenthicola* and *Thomisus nepenthephilus* [34,35]. These spiders feed on visiting insects drawn to the pitcher-plants [34,36], and in some circumstances, the spiders increase pitcher-plant prey consumption by dropping consumed prey remains into the pitchers. Interestingly, two studies by Lim et al. [34], and Lam and Tan [37] concluded that the type of association between crab spiders and pitcher-plants is environmentally context-dependent. Lam and Tan [37] demonstrated that *T. nepenthephilus* increased the prey capture rates of *Nepenthes gracilis*, offsetting the nitrogen loss from consumption by *T. nepenthephilus*, resulting in an overall net gain. However, this benefit only occurs under conditions where prey availability is low and is ultimately lost when prey availability increases, switching from a positively facilitative to a parasitic interaction [37]. 

Furthermore, a number of spider species have been reported to have unusually close associations with trichome-bearing plants [9,38,39,40,41]. One genus from the Oxyopidae family, *Peucetia*, dominates such interactions and many species are considered to have strict, and perhaps obligatory, associations with glandular trichome-bearing plants [39,40,42]. Glandular trichomes are hair-like structures believed to have evolved as a direct biotic defence against herbivorous insects [43,44]. The insects and carrion (i.e., dead insects) trapped by the glandular hairs represent an energetically cost-free, accessible food source [45], which attracts arthropod predators, such as spiders, for added protection against herbivory [40,45,46]. In three complementary studies, Morais-Filho and Romero [39,40,47] observed *Peucetia flava* exclusively in association with *Rhyncanthera dichotoma*. During the latter study, Morais-Filho and Romero [40] physically removed the glandular trichomes from *R. dichotoma* and documented fewer *Peucetia* spiders occupying those plants compared to *R. dichotoma* with intact trichomes, further demonstrating the strong and potentially obligatory association *Peucetia* spiders have with glandular trichome-bearing plants [42]. Morais-Filho and Romero [40] reported that *P*. *flava* reduced herbivory in the buds and flowers of *R*. *dichotoma* and although this interaction did not increase fruit production, it also did not incur any significant costs to *R*. *dichotoma* fitness (i.e., through predation of pollinators), signifying a potential protective mutualism. Moreover, a recent study by Sousa-Lopes et al. [45] found that the presence of *P. flava* on the trichome-bearing *Mimosa setosa* var. *paludosa* positively correlated with an increase in trapped prey and carrion. 

Spider–plant associations that arise from an exploitable source of food are not uncommon. While some spiders may associate with plants that attract and/or trap insect prey, such as glandular trichome-bearing plants and pitcher-plants, other spiders species seek nutrition from the plant itself. The salticid, *Bagheera kiplingi*, for example, is exclusively associated with many myrmecophytic acacias [7,48]. These acacias produce Beltian bodies to attract ants that protect the plant, and in return, the ants gain nutritional rewards and refuge [1,7]. The spider exploits this ant–acacia mutualism and consumes the Beltian bodies as its primary food source, which in some cases constitute 90% of its diet [48]. Therefore, it is conceivable that access to a convenient source of prey is another primary driver of spider–plant associations, and perhaps the obligatory associations observed between *Peucetia* and glandular trichome-bearing plants and Thomisidae and *Nepenthes* pitcher-plants.

Another potential driver of host plant selectivity in spiders could be crypsis (i.e., camouflage), whereby a spider may exhibit a preferential affinity for a substrate (e.g., flower, bark, and moss) that matches their body colouration/morphology, rendering them undetectable to potential predators or unsuspecting prey. Cryptic colouration is particularly well studied in Thomisidae crab spiders, which, in sit-and-wait predators, increases foraging success [49,50,51]. Certain species will preferentially select flowers, upon which they forage, that match their body colouration (i.e., background-matching) to avoid detection by pollinators and other visiting insects [41,49,52]. Moreover, there are some spider species that are also capable of changing their body colouration to match their chosen background, or in this instance, host plant. Such examples include the crab spiders *Misumena vatia* and *Thomisus onustus* that typically alternate between white and yellow [50,53]. It is evident that cryptic species will select specific substrates to ensure successful camouflage. However, there is a paucity of information to discern whether cryptic colouration is a resultant factor in specific spider–plant associations. Most crab spiders appear to be generalists, selecting a number of plant species that suit their needs.

From the examples provided above, it is particularly apparent that *Psecas chapoda* facultatively relies on the microhabitat created by *B. balansae* for foraging, mating, and oviposition, as observed by Romero and Vasconcellos-Neto [28,29], and as a refuge and nursery site that can offer protection from predators and desiccation [28,29,54,55]. Omena and Romero [56] inferred that this extreme fidelity was related to microhabitat structure, and observations by Romero and Vasconcellos-Neto [28,55] affirmed this after finding that *P. chapoda* seldom colonised bromeliads in forest habitats as leaves would often obstruct the rosette, hindering any use of the microhabitat. Likewise, some studies have reported that *Peucetia* spiders preferentially select larger plants as they offer more sites to forage and refuge, and attract and trap more insect prey [45,57]. Prey, and other sources of nutrients, are also key determinants, especially in terms of exploitable sources of food, which we see examples of in all three spider families discussed. In summary, we can infer that it is the availability of certain exploitable resources, together with a microhabitat structure and plant morphology that complements the ecological requirements, foraging the strategies and behavioural preferences of a spider [9,23,56,58,59,60,61], which are the primary factors that drive specific spider–plant associations. 

**Table 1:** The most prominent cursorial spider–plant associations. With information on the spider and host plant family and species, information on the association, and the location(s) where said interaction was documented.

Spider Family	Spider Species	Plant Family	Plant Species	Association	Region	Source
Oxyopidae	*Peucetia flava*	Asteraceae	*Trichogoniopsis adenantha*	Facultative mutualism; reduced herbivores.	Southeast Brazil	Romero et al. [46]
		Melastomataceae	*Rhyncanthera* *dichotoma*	Commensalism/facultative mutualism; protection and significantly reduced herbivory after rainy season.	Southeast Brazil	Morais-Filho and Romero [39,40,47]
		Solanaceae	*Solanum thomasiifolium*	Facultative mutualism; likely protection.	Southeast Brazil	Jacobucci et al. [57]
		Fabaceae	*Mimosa setosa var. paludosa*	Facultative mutualism; reduced exophytic herbivory, but not endophytic herbivory.	Southeast Brazil	Sousa-Lopes et al. [45]
	*Peucetia* *rubrolineata*	Asteraceae	*Trichogoniopsis adenantha*	Facultative mutualism; suppressed herbivory.	Southeast Brazil	Romero et al. [46]
	*Peucetia viridans*	Euphorbia-ceae	*Cnidoscolus* *aconitifolius*	Preference/Unknown	Southeast Mexico	Arango et al. [62]
			*Croton* *ciliatoglandulifer*	Preference/Unknown	West Mexico	Corcuera et al. [63]
Salticidae	*Bagheera kiplingi*	Fabaceae	*Vachellia spp. (myrmecophytes)*	Exploitative/Commensalism	Southeast Mexico, Northwest Costa Rica	Meehan et al. [7]
	*Evarcha* *culicivora*	Euphorbia-ceae	*Ricinus* *communis*	Unknown/Commensalism	West Kenya	Cross [64]
			*Lantana camara*	Unknown/Commensalism	West Kenya	Cross [64]
	*Pelegrina* *tillandsiae*	Bromeliaceae	*Tillandsia* *usneoides*	Obligate commensalism; strict association, but no reported costs or benefits.	Southeast USA	Young and Lockley [24]
	*Psecas chapoda*	Bromeliaceae	*Bromelia* *balansae*	Facultative mutualism; the spider aids in nitrogen acquisition.	Northeast Bolivia, Northeast Paraguay, South Brazil, Central-West Brazil	Romero [26]; Romero and Vasconcellos-Neto [29]; Romero et al. [31]; Omena and Romero [56]
Sparrasidae	*Delena melanochelis*	Myrtaceae	*Eucalyptus nitens*	Unknown/Commensalism	Australia	Agnarsson and Rayor [65]
			*E.* *regnans*	Unknown/Commensalism	Australia	Agnarsson and Rayor [65]
Thomisidae	*Misumenops* *argenteus*	Lamiaceae	*Hyptis* *suaveolens*	Unknown/Commensalism	Southeast Brazil	Romero and Vasconcellos-Neto [27]
		Asteraceae	*Trichogoniopsis adenantha*	Facultative mutualism; reduced herbivory	Southeast Brazil	Romero and Vasconcellos-Neto [27,55]
	*Misumenops* *pallidus*	Orchideaceae	*Chloraea alpina*	Commensalism	East Argentina	Quintero et al. [66]
		Ranunculaceae	*Anemone* *multifida*	Commensalism	East Argentina	Gavini et al. [41]
	*Misumenops* *nepenthicola*	Nepentha-ceae	*Nepenthes* *gracilis*	Unknown/Commensalism	North Borneo	Karl and Bauer [67]
			*N.* *rafflesiana*	Unknown/Commensalism	North Borneo	Karl and Bauer [67]
	*Synaema* *marlothi*	Roridulaceae	*Roridula dentata*	Obligate kleptoparasitism	Southern South Africa	Anderson and Midgley [38]; Anderson [68]
	*Synaema* *obscuripes*	Nepentha-ceae	*Nepenthes madagascariensis*	Unknown	Southeast Madagascar	Rembold et al. [36]
	*Thomisus* *nepenthephilus*	Nepentha-ceae	*Nepenthes* *gracilis*	Obligate, conditional facilitative mutualism	North Singapore	Lim et al. [34]; Lam and Tan [37]

### 2.2. Web-Building Spiders

Research on web-building spider–plant associations is far less numerous than on their non-web-building counterparts. Currently, there are only a few examples of exclusive spider–plant associations, represented by *Eustala* (Araneidae) and *Laetesia raveni* (Linyphiidae), which are discussed in more detail in Section 3 below. The research on cursorial spider–plant associations indicates that the suitability of a plant as a microhabitat to find shelter or food resources (i.e., prey, carrion or nectar) are the main determinants of host plant selection and subsequent spider–plant associations. This also applies to web-building species, where it is vital to select a web-building site that maximises foraging success [58]. For these sit-and-wait predators this is ultimately dependent on the density of prey [69,70], which as mentioned is a key driver in host plant selection. However, the key driver of foraging success for a web-building spider is the optimal construction of its web; hence, the majority of available research on web-building spiders documents preferential, facultative associations with plants that provide suitable structural features for web construction [48,71,72,73,74]. 

Two neotropical spider species, the theridiid *Latrodectus geometricus* and the araneid *Alpaida quadrilorata*, are both found in association with *Paepalanthus bromelioides* [73]. This rosette-structured plant provides the spiders with the structural necessities for web construction and may also offer refuge and protection from predators [23,75]. More importantly, *P. bromelioides* is considered to be a protocarnivorous plant that obtains nutrients from insects with the aid of digestive mutualists, namely *L. geometricus* and *A. quadrilorata* [73]. This plant apparently possesses features that attract insect prey, such as leaves that reflect ultraviolet light and a phytotelma (i.e., a water-filled cavity) with specialised fluid that also digests captured prey [73,76]. Similar to the pitcher-plant dwelling Thomisidae crab spiders that forage at the mouth of the pitcher, *L. geometricus* and *A. quadrilorata* build their webs above the phytotelma [76], providing easy access to incoming prey. Both spider species capture prey, while discarding carcasses and faeces into the rosette of *P. bromelioides* effectively, and thereby channelling a more bioavailable form of nitrogen directly to the plant [31]. Nishi et al. [73] observed *A. quadrilorata* strictly on *P. bromelioides* within the study area in Morro da Pedreira, Brazil. However, no other research is available to determine how exclusive this association is, and since *L*. *geometricus* has been documented on other plant species (e.g., [68]), both should be considered facultative digestive mutualists. 

As previously discussed, carnivorous plants present spiders with a suitable microhabitat [34,37]. However, aside from Nishi et al. [73], there are no reports of unequivocal web-building spider associations with carnivorous plants. Cresswell [77] observed an unidentified species of linyphiid occupying the pitcher-plant *Sarracenia purpurea* as an apparent kleptoparasite. Milne and Waller [78] similarly observed linyphiids interacting with *S. purpurea*, using the pitchers as substrates to build their horizontal sheet webs. However, Milne and Waller [78] noted that many of the linyphiids constructed their webs at a height similar to the pitchers, implying that this a spatial coincidence rather than an association. The theridiid *Theridion decaryi* has also been observed inhabiting a different pitcher-plant species, *Nepenthes madagascariensis*, according to Fage [79]. The available research on these interactions is evidently scarce and ambiguous. However, considering that several other spider species have been found in association with pitcher-plants and other carnivorous plants (Table 1), the possibility that there are species of web-building spiders closely associated with pitcher-plants cannot be ruled out.

In addition, web-building spiders in the genus *Stegodyphus* (Eresidae) have strong affinities for thorny plants [54,72,80]. A recent study by Rose et al. [54] determined that *Stegodyphus dumicola* nests occurred more frequently on tall thorny plants and were observed on several different genera. Lubin et al. [80] also found that *S*. *lineatus* preferred to inhabit tall, thorny, and even poisonous plants. Thorny plants offer protection against predators (e.g., birds) and reduce the risk of disturbances from large herbivorous animals (e.g., cattle and other browsing/grazing mammals) that can damage or destroy spider webs [54,72,75,80]. Ruch et al. [72] demonstrated that *S*. *tentoriicola*, which inhabits both thorny and thornless plants, constructed larger webs when inhabiting thorny plants, and were less likely to relocate, compared to spiders in thornless vegetation. As larger webs are more costly to build, it is evident that thorny plants provide *S*. *tentoriicola*, and likely other spider occupants, with favourable microhabitats that enable spiders to invest more energy into building larger webs, increasing their foraging success, whilst receiving refuge and protection from animal-related disturbances [54,72].

Extreme specificity and fidelity toward host plants is evidently not as common among web-building spiders. Many web-building spiders often interact with and inhabit multiple plant species from different families and orders, as described, for example, by Rose et al. [54] and Whitney [71]. A recent study conducted by Cuff et al. [81] in England evaluated the leaf and habitat preferences for oviposition in the candy-striped spiders *Enoplognatha ovata* and *E. latimana* in the family Theridiidae. These spiders create a retreat, or nest, for oviposition by rolling a leaf with silk [81]. *Enoplognatha* appeared to preferentially select the leaves of bramble (*Rubus fruticosus*), nettle (*Urtica dioica*), hogweed (*Heracleum sphondylium*), and have also been found using fireweed (*Chamaenerion angustifolium*) for their leaf-roll nests. Plant preferences were not taxon-related, nor was the size and structure of leaves important; however, certain traits, such as the length–width ratio, were thought to influence leaf selection [81]. Cuff et al. [81] even suggested that the spiders could possibly provide a degree of protection from herbivorous insects in a mutualistic association. 

## 3. Host Plant Specificity in Web-Building Spiders: The Unique Cases of *Eustala* and *Laetesia*

Very few one-to-one obligatory associations between spiders and specific plants have been described and, as mentioned above, most of these involve cursorial spiders. Here, we discuss the two examples we found: one in the orb-web genus *Eustala* (family Araneidae) and one in the sheet-weaver *Laetesia raveni* (family Linyphiidae).

### 3.1. The Araneid Orb-Web Spiders in the Genus Eustala

Species in the orb-web family Araneidae commonly inhabit plants on which they construct their webs. Although none of the genera in this large family are characterised as being closely associated with particular plant groups or plant species, recent work indicates that several species in the genus *Eustala* exhibit varying degrees of host plant specificity. The genus *Eustala* is large with around 90 species distributed throughout North and South America, the majority of which are found at tropical latitudes [82,83,84]. Early studies of the natural histories of the *Eustala* species noted that they do not typically build a retreat but rather rest on branches or are tucked into dead vegetation that they resemble in colour and pattern near their webs (e.g., [85,86]).

*Eustala perfida*, for example, exhibits a colour polymorphism that closely resembles the mosses and lichens on the tree trunks on which it builds its webs in semi-deciduous rainforests in south eastern Brazil. A detailed study of spatial distribution and substrate selection showed that this spider apparently prefers specific microhabitats characterised by large-diameter rough-barked trees with mosses, lichens, and concavities, but that it does not uniquely inhabit the bark of any one particular tree species [87]. Two other *Eustala* species in south eastern Brazil, *E. sagana* and *E. taquara*, however, show a closer association with particular plant species. Both spider species preferentially rest on dead vegetation, against which they are strongly camouflaged, versus live vegetation (see images in Souza et al. [88]). A comparison of the relative frequencies of plant species in the spiders’ preferred edge habitats with the relative frequencies of plant species used for web construction provides evidence for some level of host plant specificity. *Eustala taquara* occupied the fleabane *Conyza bonariensis* significantly more frequently than other plant species, whereas *E. sagana* significantly more frequently occupied a different weedy plant species, *Hyptis suaveolens* [88]. Preferential use of these plant species for web construction and retreats may reduce conflict between the two spider species in an area of range overlap along an elevation gradient [88].

**Figure 1:**
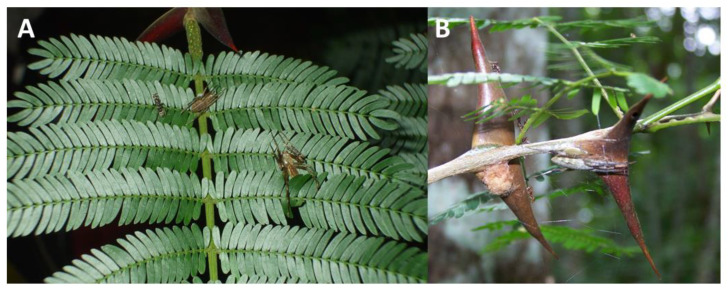
(**A**) A female (left) and (**B**) male (right) *Eustala oblonga* on the foliage of *Vachellia melanoceras* in Parque Nacional Soberania, Panama. (**A**) An adult female *E. illicita* and her egg sac on *V. collinsii* near Madden Dam, Panama. Note the patrolling acacia ants on the leaflets and the thorns.

**Figure 2:**
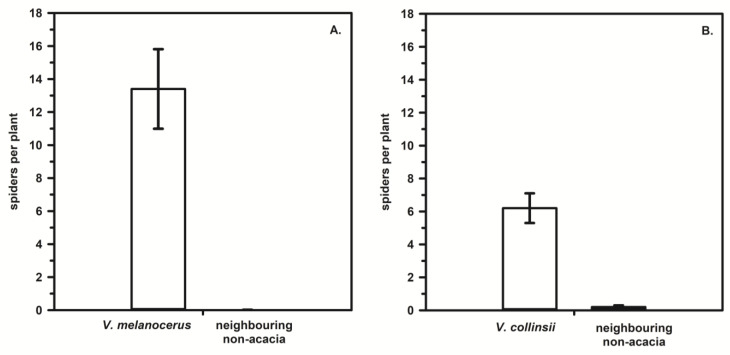
(**A**). *Eustala oblonga* abundance on *Vachellia melanoceras* acacias and randomly selected neighbouring non-acacias in Parque Nacional Soberania, Panama (ltwo sample t-test: t_98_ = 10.97, *p* < 0.0001). (**B**). *Eustala illicita* abundance on *V. collinsii* acacias and randomly selected neighbouring non-acacias in Parque Natural Metropolitano, Panama (Mann–Whitney U test: U_18_ = 5.6, *p* < 0.0001 from Hesselberg and Triana [11]). Error bars in both panels represent the standard error.

The evidence for even stronger associations between *Eustala* spiders and specific plant species comes from research in central Panama. *Eustala oblonga* and *E. illicita* are found in abundance on the ant acacias *Vachellia melanoceras* and *V. collinsii* on the Atlantic and Pacific sides of the Continental Divide, respectively [11,12,89]. Remarkably, on plants on which patrolling acacia ant mutualists tolerate few other animal interlopers, these two spider species construct webs at night and rest by day on the acacia leaves, branches, and thorns, where they are mostly ignored (or undetected) by the ants; they also breed and construct egg sacs on the acacias (Figure 1). Neither *E. oblonga* nor *E. illicita* prey on patrolling ants, but they do capture dispersing acacia ant alates in their webs in addition to many other flying insects [89]. Surveys of 50 *V. melanoceras* acacias, 50 neighbouring non-acacias (J.D. Styrsky and J.N Styrsky, unpublished data), 18 *V. collinsii* acacias, and 18 neighbouring non-acacias [11] showed that both *E. oblonga* and *E. illicita*, respectively, are found almost exclusively on ant acacias (Figure 2). Although neither spider is typically encountered elsewhere in the forest understory, a few individuals of both species were observed resting on dead, weedy, roadside vegetation in Parque Nacional Soberania and Parque Natural Metropolitano, respectively (T. Hesselberg and J. Styrsky, unpublished observations), raising the possibility that their association with ant acacias may not be entirely obligatory.

Despite whether or not *E. oblonga* and *E. illicita* are truly host-plant-specific, they are seemingly adapted to inhabiting ant-defended acacias. Patrolling acacia ants regularly encounter the spiders as they rest on the plant surface, often stopping to antennate them before moving on, unperturbed. The spiders typically refrain from reacting to the ants even if the ants walk directly over them. An experiment comparing the reaction of *Pseudomyrmex satanicus* ants on *V. melanoceras* to active versus immobilised *E. oblonga* spiders showed that immobilised spiders did not elicit an aggressive response in the ants. Moving spiders, however, immediately incited ants to become agitated and attack [12]. In response to ant aggression, the spiders either retreated to web strands or, more frequently, ran a short distance and then stopped and crouched against the plant surface, thereby preventing detection by the ants. In contrast, another araneid orb-web species from the surrounding understory used in this experiment, *Argiope argentata*, reacted quite differently. If they were confronted by patrolling ants, instead of running a short distance and then sitting still, they continued to run, further stimulating ant aggression until they were killed or forced off the plant [12]. What do these spiders gain by inhabiting plants patrolled by dangerous ants? In a field experiment in which entire acacia ant colonies were removed from *V. melanoceras* acacias, the abundance of *E. oblonga* spiders decreased significantly over time compared to control acacias. Concomitantly, the abundance of natural enemies of spiders increased on the acacias from which ants were removed, perhaps because they were no longer deterred by patrolling ants. These results suggest *E. oblonga* spiders may be adapted to exploit their hosts’ ant–acacia mutualism for enemy-free space [13].

Besides employing behavioural mechanisms to avoid ant aggression, *E. oblonga* and *E. illicita* may also mask their presence on the acacias chemically, either by synthesising or absorbing odours into their cuticles of the *Pseudomyrmex* ant mutualists or their host acacias. Chemical mimicry of host ants has been documented in spider myrmecophiles in a few families that are either predators of ant larvae or kleptoparasites of ant prey (reviewed in Cushing [90]), but such an interaction has not been documented for any araneid spider. Bolas spiders, which are web-less spiders in the family Araneidae, demonstrate that araneid spiders can use chemical mimicry as they emit volatiles that mimic the pheromones of female moths to lure the males close so that they can catch them with their bolas [91]. 

**Figure 3:**
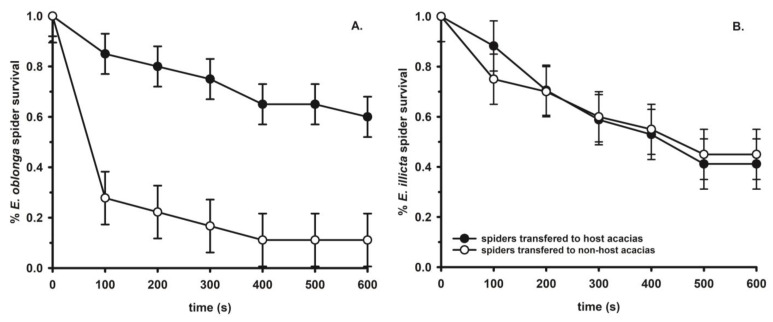
Results of Cox proportional hazards failure-time analyses comparing the percentage of survival over time. (**A**). *E. oblonga* spiders transferred from their home host plant (*V. melanoceras*) to either another host acacia or a non-host acacia (*V. collinsii*) (*X*_1_^2^ = 15.41, *p* < 0.0001). (**B**). *Eustala illicita* spiders transferred from their home host plant (*V. collinsii*) to either another host acacia or a non-host acacia (*V. melanoceras*) (*X*_1_^2^ = 0.21, *p* = 0.65). These experiments were conducted in Parque Nacional Soberania and Parque Natural Metropolitano, Panama in 2008. Error bars in both panels represent the standard error.

A preliminary investigation of the chemical mimicry hypothesis provides conflicting evidence for this. In a translocation experiment (K. Marvin and J.D. Styrsky, unpublished data), freshly killed *E. oblonga* and *E. illicita* spiders were moved to either a different individual of their own host acacia species or to non-host acacia species across the Panamanian isthmus, and the time until the spiders were attacked and dragged off the foliage was recorded. The spiders were frozen immediately before being placed on acacias to isolate any effect of chemical camouflage from spider movement that might stimulate ant aggression. Failure-time analyses showed that *E. oblonga* spiders were attacked by patrolling ants significantly more rapidly on non-host acacias (*V. collinsii*) than on their own host acacias (*V. melanoceras*) (Figure 3A). Further, patrolling ants were significantly more likely to lunge at (a confrontational encounter but not an actual attack) *E. oblonga* spiders on non-host acacias than on their host acacias. These results could suggest that *E. oblonga* spiders were ‘chemically familiar’ to the ants on the spiders’ host acacias, but that the ants on the non-host acacias perceived *E. oblonga* spiders as foreign. Contradictory to these results, however, *E. illicita* spiders were no more likely to be lunged at, and were attacked no more frequently on non-host acacias (*V. melanoceras*) than on their host acacias (*V. collinsii*) (Figure 3B). These results are difficult to interpret. At this point, the cuticular chemistry of neither the spiders nor the ants has been analysed to further investigate the chemical mimicry hypothesis.

The cues *Eustala oblonga* and *E. illicita* use to find and discern their respective host acacias from the surrounding understory vegetation are also currently unknown. *Vachellia melanoceras* is sparsely distributed within its range on the Atlantic side of central Panama [89], potentially making it difficult to target. Despite this low density, mature *V. melanoceras* acacias (10–15 m in height) can host hundreds of adult *E. oblonga* spiders (J.D. Styrsky, unpublished data). *Vachellia collinsii* can occur in greater densities in the Pacific side of Panama, but it depends on the particular site [89]. Previous work shows that spiders that are associated with plants use visual, olfactory, and tactile cues to locate specific plant species (reviewed in Vasconcellos-Neto et al. [9]). Given that some spiders are sensitive to plant volatiles, as discussed above, it is possible that *E. oblonga* and *E. illicita* use volatiles produced by the acacias or their acacia ant mutualists to locate host acacias. In a simple choice experiment (D. Clement and J.D. Styrsky, unpublished data), adult *E. oblonga* spiders were offered freshly collected foliage of *V. melanoceras* in one 15.5 cm diameter tube chamber and freshly collected foliage from another understory woody plant haphazardly selected from the immediate vicinity of the acacia in a second chamber. The same experiment was set up to test *E. illicita* spiders using the foliage of its host plant, *V. collinsii*. Individual spiders were placed in a shorter and narrower tube in between the two plant chambers and left for twelve hours. In 13 out of 16 trials, *E. oblonga* spiders were found occupying the acacia foliage (i.e., not just in the chamber with the foliage). Similarly, in 14 out of 16 trials, *E. illicita* were found occupying the acacia foliage. In both experiments, acacia ants had been removed from the acacia foliage before placing the foliage in the chambers, but their cuticular hydrocarbons might still have been detectable.

### 3.2. The Linyphiid Laetesia Raveni

**Figure 4:**
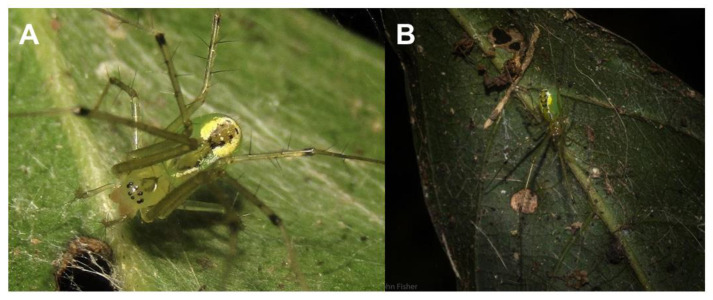
Photographs of *Laetesia raveni* (Linyphiidae) showing its close association with vegetation. (**A**). Frontal view. Photo taken by Samuel Frankel from iNaturalist. (**B**). Dorsal view demonstrating flattening against the leaf of *Mallotus philippinensis* in a possible camouflage attempt. Photo courtesy of Nick Fisher from Flickr (dustaway).

Examples of close spider–plant associations among linyphiids and other web-building families, such as Theridiidae, are rare, and often those described as such do not hold up to closer scrutiny. For example, aside from the previously mentioned *Latrodectus geometricus* (see Nishi [92]; Nishi et al. [73]), *Dipoena banksii* is the only other theridiid reported to have a close plant association. This species is commonly found on *Piper* plants indirectly through its preferred ant prey, which exclusively inhabits *Piper* species [93]. Research is especially limited with regard to linyphiids, and most available accounts of linyphiid–plant interactions are inexplicit, such as the observations by Cresswell [77] and Milne and Waller [78], and a study by Bomfim et al. [75], which recorded an association of two Erigoninae linyphiids with the thorny rosette-structured plant *Eryngium horridum*. Thorny plants can provide important microhabitats for some web-building spider species, as demonstrated by Ruch et al. [72] and Rose et al. [54]. The thorns create a complex architecture that provides sufficient web attachment sites whilst simultaneously protecting the spiders from disturbances or threats [54,58,72,74]. Interestingly, a new species of Linyphiidae, *Laetesia raveni* (Figure 4), recently described by Hormiga and Scharff [10], has been observed exclusively on two thorny plant species, *Calamus muelleri* and *Solanum inaequilaterum* in Queensland, Australia. The unique case of *L*. *raveni* is currently the only recorded instance of a linyphiid exhibiting host plant specificity. This linyphiid constructs a dome-shaped web on its chosen host plant, and according to Hormiga and Scharff [10], the spiders were typically situated upside-down on the web, directly under a leaf that was positioned at the centre of the web. Often, *L*. *raveni* was observed flattening its body against the leaf when provoked. The authors suggest that this behaviour, combined with the spider’s unique green colouration, is a form of crypsis (Figure 4). *Laetesia raveni* was more common on *C*. *muelleri*, a climbing palm with stems densely covered with thorns, and with the leaflets, stalks, and midribs of the fronds also bearing small spines. Similarly, *S*. *inaequilaterum* has thorns or spines covering the stem and leaves; however, the thorns are much denser along the stem. There have been two recorded instances where *L*. *raveni* was found on other undocumented plant species, but these plants were seemingly in physical contact with either *C*. *muelleri* or *S*. *inaequilaterum* [10]. The ecology of *L*. *raveni* and its unusual association with *C*. *muelleri* and *S*. *inaequilaterum* requires considerably more research, especially to: (1) Determine if this is a host-specific association (unpublished observations from the rainforest reserve in Lismore, New South Wales suggest that it can be found on other plant species (N. Fisher, personal communication October 2022, Figure 4)); (2) Further understand whether *L*. *raveni* inhabits the host plants for protection from natural enemies, as seen in *Stegodyphus* species (see Ruch et al. [72]; Rose et al. [54]); and (3) Determine if the green colouration and body-flattening behaviour are forms of passive predator defence (i.e., crypsis).

## 4. How Might Spiders Identify and Locate Their Host Plant?

### 4.1. Website Choices and Web Building

Web-building behaviour is relatively well studied, especially in tangle-(Theridiidae) and orb-web spiders (Araneidae and Tetragnathidae), where it follows a generally rigid pattern of stereotypic behaviours, although with some flexibility [94,95]. On the other hand, however, we still know very little about the process of habitat exploration and site selection that precedes web construction [96,97]. Orb-web spiders engage in extensive site exploration [98], and generally match the shape of their webs to both the available space [99] and the available silk supplies [100]. Most web-building spiders are not picky when it comes to attaching their webs to their surroundings and will choose any suitable structure—usually a rigid or semi-rigid structure in order to avoid web damage from wind movements [5,101,102]—although some spiders also attach their webs to moving structures, such as leaves and grass, without it negatively affecting their web-building efficiency or resultant webs [103]. Linyphiid spiders in grassland, for example, do not show any preference for specific plant species, but consistently select tall and stable vegetation to attach their webs to [5]. Similarly, the desert-inhabiting social eresid spiders, *Stegodyphus dumicola*, construct their colonial webs on taller rigid plants with thorns [54]. Individual spiders seem to select optimal host plants based on the structural properties of the plant, including their fractal dimension [104], while some web-building spiders select web-attachment sites on substrate depending on its hydrophobicity [105].

We know virtually nothing about how the few web-building spiders with specific host plants choose them, but interestingly even these associations can be flexible. For example, as discussed above, the acacia orb-web spider *Eustala illicita* is almost exclusively found on the acacia *Vachellia collinsii* with only four juveniles out of a total of 117 observed spiders found in neighbouring vegetation. It nonetheless readily builds webs in sterile plastic frames in the laboratory [11]. All age classes, from early juveniles to adult females, build webs in captivity at high web-building frequencies with the webs being, at least superficially, very similar to the ones built in the wild [11,106]. Spiders in the lab also show a high degree of flexibility in adapting their web shape to differently shaped plastic frames [99]. No learning seemed to be involved as the second and third webs constructed in the frames are no different than the first web [107], which suggests that this flexibility is regularly needed in their natural habitats. This fits well with the observation that acacia spiders can be found in high densities on their acacia host plants [13], which presumably gives rise to competition over suitable web-building spaces forcing some spiders to build at less optimal sites within the tree, where adaptations to the standard orb web shape and structure are required. In the case of the web-building spiders that are not closely associated with specific species of plants, and possibly also for those few that are, suitably structured vegetation for building webs is probably found by random searching and mechanical contact stimulation, as web-building spiders typically have very poor vision [108]. 

Many spiders engage in random dispersal through ballooning either as adults (if small spiders) or in the early juvenile stages. This involves releasing silk threads into the wind, where a combination of electric and aerodynamic forces lift the spider into the air and potentially disperse it over long distances [109,110]. Ballooning is common in web-building spiders (e.g., [111,112,113,114]). Ballooning propensity is highest in spiders living in open ecosystems, although one study found that some spiders from temperate woodlands can have high ballooning propensity similar to those of grassland [115]. To our knowledge, however, no data are available on ballooning propensity in web-building spiders in tropical rainforests, so it remains currently unknown if spiders associating with specific tropical forest trees, such as the acacia orb-web spiders in the genus *Eustala,* discussed in this paper, use short distance ballooning as a host plant location strategy. 

Spiders are known to use short-distance random or systematic search strategies for locating lost egg sacs and caught prey with examples of the former from cursorial spiders [116] and the latter from web-building spiders [117,118]. On the other hand, while some spiders are known to be able to find their burrows over long distances, likely using compass and path integration [119,120], no information, at least not to our knowledge, is available on the extent to which spiders rely on random-search patterns to find suitable web-building sites over longer distances. However, the hypothesis that many spiders engage in random searching and potential trial-and-error web-building behaviour on chosen sites is strengthened by the observations that some orb-web spiders, despite extensive site exploration prior to web-building [97], build a smaller explorative web when building at a new location [98], and readily move their webs when encountering low prey capture, or when suffering web damage [70,121,122].

### 4.2. The Use of Chemical Cues and Communication in Spiders

The alternative to the random or systematic search strategies for finding suitable plant hosts discussed above is a more targeted strategy using chemical cues. Spiders are known to use chemical cues in sexual communication, especially in relation to males locating females through silk-borne [123] or cuticular cues [124]. We refer readers to the recent excellent review by Fischer [19] on chemical communication in spiders focusing on a methodological overview on how to study their pheromones. Spiders can also detect predators such as ants through semiochemicals [125], and they are sensitive to the chemical cues of potential predators [126,127]. The wolf spider *Pardos milvina*, for example, alters where it forages when it chemically detects one of its predators, the larger wolf spider *Trigrosa helluo* [126]. 

Many spiders associated with ants use chemical cues from the ants to prey upon them. The mimicry of cuticular hydrocarbons (CHCs) is recognized as one of the most common mechanisms that myrmecophiles and termitophiles use to deceive their host [128] but evidence from spiders is scarce. The jumping spider *Cosmophasis bitaeniata* uses the CHC mimicry of its ant host *Oecophylla smaragdina* to prey on larvae [129]. Interestingly, the spider does not acquire the chemical mimicry by physical contact with the adult ants, but it acquires it from eating the larvae, and the variation in CHCs profiles across spiders is colony-specific [130]. Another foraging strategy is chemical eavesdropping, as in the myrmecophagous jumping spider *Habrocestum pulex,* which initiates predatory behaviours when presented with airborne and soilborne chemical cues from the ants [21]. Chemical eavesdropping can show phenotypic plasticity, as seen in the jumping spider *Cyrba algerina*, which varies its responsiveness towards spider prey odours depending on whether the prey species cohabits with the spider or not [131]. Eavesdropping can also be used by ant-mimicking spiders to find their mimetic model ant species (Batesian mimicry) without preying on the ants [132]. In other instances, spiders seem to choose a habitat that increases their chances of foraging success. For example, the western black widow *Latrodectus hesperus* prefers to build its webs in microhabitats where it detects the residual chemical cues of house crickets [133].

As discussed in the ‘spider–plant associations’ section above, spider–plant interactions are now widely described and plants with rosette-shaped clusters of leaves or tri-chomes are the most common plant architectures to have associations with spiders [9]. The evidence that some spiders select plants with similar architectural features by using visual cues is strong (reviewed in Vasconcellos-Neto et al. [9]), but few studies have explored whether chemical signals are involved in host plant recognition. However, the examples are known to include the nursery web spider [134], crab spiders [22,135], and jumping spiders [136]. For instance, pitfall traps baited with eugenol—which is a flower component fragrance—caught more individuals of two *Thomisus* species (Thomisidae), as compared to controls [22]. Similarly, *Thomisus spectabilis* chose the same flower more often than a honeybee, when there was a flower odour signal present [135]. Interestingly, chemical recognition of the host plant is species-specific in some cases, with some plant chemicals inducing responses in some spiders but not in others [134,137]. Thus, it remains a possibility that the *Eustala* spiders use plant volatiles to locate their hosts.

## 5. Conclusions and Future Directions

We found, despite extensive literature searches, only two examples of web-building spiders showing host specificity, and even in these two examples, some individuals were observed on non-host plants. Our study, therefore, suggests that web-building spiders in general are less likely to form one-to-one associations with specific species of plants than cursorial spiders (see also Vasconcellos-Neto et al. [9]). One reason for this could be due to a lack of research focusing on looking for these relationships in web-building spiders, but it is likely that they are in fact rare since web-building spiders create their own modified microhabitat with the web acting like an extended phenotype [138]. If the surrounding vegetation is reduced to just providing support or shelter for the web [97,139], it stands to reason that web-building spiders have a far less intimate relationship with the vegetation than the cursorial spiders that spend most of their life in direct contact with one or a few species of plants. 

More studies on host plant and web-site preferences are needed in web-building spiders, especially of smaller spiders, such as many linyphiids and some theridiids, which construct small webs fully within a single plant. Undoubtedly, more examples of host specificity in web-building spiders await discovery, especially from tropical regions where the diversity of these spiders is highest. We also need more detailed studies on the known interactions as many unanswered questions remain, including whether spiders that use several host plant species [57,61] rely on the physical attributes of the plants or if instead these plants share similar chemical profiles or particular molecules that facilitate recognition. Spiders that specialise in certain plant families as host plants [61] are good candidates to address these questions. Furthermore, ontogenetic variation should be integrated into the study of spider chemical ecology. For instance, plant specialisation in the Japanese foliage spider, *Cheiracanthium japonicum*, seems to develop with age, with juveniles and adults using different plant species in some cases [140].

In the present study, we found one very interesting and well-evidenced example coming from the acacia orb-spiders in the araneid genus *Eustala*, which seem to use the acacia and their ant protectors for enemy-free space [13] without causing any significant harm to either the plants or the ants [89]. The particularly interesting aspect of this example is that we currently know of two species of *Eustala* that are associated with two different species of *Vachellia* with their own specific species of *Pseudomyrmex* ants [11,12]. While a few individuals have been found in nearby vegetation, particularly in dead vegetation as is common in other species of *Eustala* [86,88], this indicates a high degree of host specificity, probably aided by a combination of behavioural and chemical mimicry to avoid attacks from the resident ants [12]. These findings and the preliminary data we discuss in Section 3.1 above suggest that both *Eustala* species may use chemical cues to discern host acacias. However, to confirm this hypothesis, larger and more detailed studies on both short-distance (centimetre scale) chemical attraction in laboratory behavioural assays with Y- or T-mazes, and longer distance (meter scale) navigation in the laboratory and in the field are needed to determine the potential role of plant volatiles and/or ant pheromones for host location identification. Similarly, we need a combination of behavioural and cuticular chemistry studies (such as comparing surface chemistry profiles in spiders, ants, and acacias with GG-MS) to determine the degree to which *E. illicita* and *E. oblonga* rely exclusively on measured behavioural responses [12] to avoid getting attacked by the aggressive *Pseudomyrmex* ants.

The scattered distribution of acacias within the rainforest could also suggest that they can be viewed as habitat islands from the perspective of the spiders and insects that utilise the ant–acacia system [141]. Thus, studies on the mechanisms behind targeted navigation and host-finding mechanisms could be combined with studies on gene flow between spider populations on individual trees or groups of trees in different parts of the same forest. DNA sequence differences, usually from mitochondrial genes, can be used to determine pairwise F_ST_ differences among samples collected at different geographic scales [142]. This method has been successfully used with orb-web spiders several times, including Lee and co-workers’ [143] study revealing a high level of gene flow between *Nephila pilipes* populations across a mountain range in Taiwan. The surprisingly high interconnectedness between these spatially isolated populations is almost certainly caused by long distance dispersal via ballooning, which many spiders engage in [144]. It is currently not known if, or to what degree, *Eustala* orb spiders engage in ballooning, but studies on the propensity of ballooning, which can easily be quantified in the laboratory [112], could be fruitfully combined with studies on gene structure to further cast light on the intimate relationships between these spiders and their host plants. 

The wide range of questions that can be asked and answered in the spider–ant–acacia system indicate that this system makes for an ideal model system for evolutionary and ecological studies, especially as comparative studies can be conducted on different closely related species and because strategies can be contrasted with other arthropods that utilise the swollen thorn acacias for enemy-free space, or engage in parasitic interactions with either the ants or the acacias [145,146].
